# Hepatitis E virus sequences in swine related to sequences in humans, The Netherlands.

**DOI:** 10.3201/eid0706.010608

**Published:** 2001

**Authors:** W H van der Poel, F Verschoor, R van der Heide, M I Herrera, A Vivo, M Kooreman, A M de Roda Husman

**Affiliations:** Microbiological Laboratory for Health Protection, National Institute of Public Health and Environment, Bilthoven, The Netherlands. Wim.van.der.Poel@rivm.nl

## Abstract

Hepatitis E virus (HEV), a major cause of viral hepatitis in much of the developing world, has recently been detected in swine in North America and Asia, raising concern about potential for zoonotic transmission. To investigate if HEV is commonly present in swine in the Netherlands, pooled stool samples from 115 swine farms and nine individual pigs with diarrhea were assayed by reverse transcription-polymerase chain reaction (RT-PCR) amplification. HEV RNA was detected by RT-PCR and hybridization in 25 (22%) of the pooled specimens, but in none of the individual samples. RT-PCR amplification products of open reading frames 1 and 2 were sequenced, and the results were compared with published sequences of HEV genotypes from humans and swine. HEV strains from swine in the Netherlands were clustered in at least two groups, together with European and American isolates from swine and humans. Our data show that HEV in swine in the Netherlands are genetically closely related to HEV isolates from humans. Although zoonotic transmission has not been proven, these findings suggest that swine may be reservoir hosts of HEV.


### *Hepatitis E Virus* Sequences in Swine Related to Sequences in Humans, the Netherlands

**Wim H.M. van der Poel,* Froukje Verschoor,* Reina van der Heide,* Maria-Inmaculada Herrera,† Amparo Vivo,† Marlou Kooreman,* and Ana Maria de Roda Husman***

*National Institute of Public Health and the Environment (RIVM), Bilthoven, the Netherlands; and †Instituto de Salud Carlos III, Majadahonda, Madrid, Spain

*Hepatitis E virus* (HEV), a major cause of viral hepatitis in much of the developing world, has recently been detected in swine in North America and Asia, raising concern about potential for zoonotic transmission. To investigate if HEV is commonly present in swine in the Netherlands, pooled stool samples from 115 swine farms and nine individual pigs with diarrhea were assayed by reverse transcription-polymerase chain reaction (RT-PCR) amplification. HEV RNA was detected by RT-PCR and hybridization in 25 (22%) of the pooled specimens, but in none of the individual samples. RT-PCR amplification products of open reading frames 1 and 2 were sequenced, and the results were compared with published sequences of HEV genotypes from humans and swine. HEV strains from swine in the Netherlands were clustered in at least two groups, together with European and American isolates from swine and humans. Our data show that HEV in swine in the Netherlands are genetically closely related to HEVs isolates from humans. Although zoonotic transmission has not been proven, these findings suggest that swine may be reservoir hosts of HEV.

*Hepatitis E virus* (HEV) is a nonenveloped RNA (7.5-kb) virus, previously classified as a calicivirus but provisionally classified in a separate family of HEV-like viruses (1). HEV is responsible for large epidemics of acute hepatitis and sporadic cases in southeast and central Asia, the Middle East, parts of Africa, and Mexico. Few HEV infections have been reported in nontravelers in industrialized countries, including the Netherlands (2). HEV infection spreads by the fecal-oral route, usually through contaminated water. The clinical illness resembles other forms of acute viral hepatitis, with onset after an 1- to 8-week incubation period. Clinical attack rates are the highest among young adults. In younger age groups, infections are more often anicteric and asymptomatic. Chronic HEV infection has not been observed. Although the death rate is usually low (0.07% to 0.6%), the illness may be particularly severe among pregnant women, with death rates as high as 25% (3). To date, no specific treatment is available for HEV infection. Ensuring a clean drinking water supply remains the best preventive strategy.

Viral excretion begins approximately 1 week before onset of illness and persists for nearly 2 weeks; viremia can be detected during the late phase of the incubation period and in the acute phase of illness (3,4). Long-term persistence of HEV in the body fluids of infected persons seems to be an unlikely reservoir for transmission of HEV (3). Experimental HEV infection in swine has been reported (5), and serologic evidence for HEV infection in swine from areas endemic for human HEV has also been reported (6). Recent isolation of a swine virus resembling human HEV suggests the possibility of zoonotic HEV infection (7).

The objective of this study was to investigate if HEV is prevalent in swine in the Netherlands and to determine the relationship between the strains detected in pigs and those described in humans.

### Methods

### Fecal Specimens

Stool specimens from swine were collected as part of ongoing surveillance for potential zoonotic microorganisms associated with gastroenteritis in humans (8). From October 10, 1998, through April 21, 1999, fecal samples were collected from 115 pig farms located throughout the Netherlands. Pig samples were collected from fattening pigs 3 to 9 months of age; farm sizes ranged from 22 to 1,600 animals. Individual stool samples were collected from nine pigs with diarrhea.

### Sampling

The sampling strategy was designed to allow monitoring for the presence of pathogens in a large number of animals; it allows detection of microorganisms at the farm level with a prevalence of 5% and 95% confidence (8). Pig farm samples were collected from animals housed in one randomly chosen farm building. A minimum of 20 and a maximum of 60 fresh stool specimens were collected per farm and pooled samples were designated as the farm sample. Fecal samples were stored until testing at -70°C in 15 g/L of Trypton Soya broth (Oxoid CM 129) and 10% glycerol.

Address for correspondence: Wim H.M. van der Poel, Microbiological Laboratory for Health Protection, National Institute of Public Health and the Environment (RIVM), Antonie van Leeuwenhoeklaan 9, 3720 BA Bilthoven, the Netherlands; fax: 3130-274-4434; e-mail: Wim.van.der.Poel@rivm.nl

### Molecular Detection of HEV

For extraction of viral RNA, stool samples were resuspended in Hanks balanced salt solution (Gibco BRL, Breda, the Netherlands) to a final concentration of approximately 10%. These suspensions were centrifuged at 3,000 x *g* for 20 minutes, and 100 µL was used for RNA extraction. Viral RNA was extracted by binding to size-fractionated silica beads (Sigma, Roosendaal, the Netherlands) in the presence of guanidinium isothiocyanate (GuSCN). Bound RNA was washed and eluted as described (9).

To reduce risk of contamination, one water sample for every four stool specimens was included as a negative control, treated the same way as the fecal samples. For positive controls, three HEV-positive samples (10% fecal suspensions) were used. One human (US-2) and one swine (Meng isolate) HEV-positive sample, both isolated in the United States, were included. The human isolate (US-2) was passaged once in a Cynomolgus macaque and once in a Rhesus monkey (*Macaca mulatta*). The swine isolate was passaged once in a Rhesus monkey. The third positive control sample was a Burmese HEV swine isolate (10), which was passaged once in Cynomolgus monkeys (*M. fascicularis*). Extraction, preparation of master mixes and reactions, and analysis of polymerase chain reaction (PCR) products were done in different rooms with designated sets of pipettes. To avoid false-positive PCR results, the precautions described by Kwok and Higuchi (11) were strictly followed.

We used single-round and nested reverse transcription (RT)-PCR assays with primer pairs, as described by Meng et al. (7), Wang et al. (12), and Schlauder et al. (13). Two of these primer pairs target a section of the open reading frame (ORF)1 gene coding for nonstructural proteins (Table 1). Three primer pairs target the ORF2 part of the HEV genome that codes for the viral structural proteins (Table 1). Primers ORF2-s1 and ORF2-a1 were used for screening and detecting HEV RNA in all fecal samples. This single-round RT-PCR amplifies 197 nucleotides of ORF2. Primer sets ORF1-s1/ORF1-a1 with ORF1-s2/ORF1-a2 and 3156-EF/3157-ER with 3158-EF/3159-IRS were used for nested PCR amplification of specific parts of the ORF1 and ORF 2 encoding regions. Second-round internal primers amplify the 286 and 348 nucleotides of ORF1 and ORF2, respectively.

For RT, 5 µL of RNA was mixed with 4 µL of 45 pmol antisense primer (ORF1a1 for ORF1 and 3157ER for ORF2). The solution was heated to 95°C for 2 minutes, and after cooling on ice, 6 µL of RT buffer was added. The RT reaction was performed in a final volume of 15 µL consisting of 10 mM Tris-HCl (pH 8.3), 50 mM KCl, 3 mM $MgCl_2$, 1 mM each of deoxynucleoside triphosphates (dNTPs), and 5 U of *avian myeloblastoma virus*-RT (Boehringer Mannheim, Almere, Netherlands). The mixture was incubated for 1 hour at 42°C, heated for 5 minutes at 95°C to denature the enzyme, and then placed on ice. Five microliters of the RT mixture was added to the PCR mix, which contained 10 mM Tris-HCl (pH 9.2), 75 mM KCl, 1.5 mM $MgCl_2$, 0.2 mM dNTPs, 2.5 units AmpliTaq (Perkin Elmer, Nieuwerkerk a/d IJssel, Netherlands), and 15 pmol sense primer (ORF1-s1 for ORF1 and 3156 EF for ORF2). The final volume of the PCR reaction was 50 µL. Mineral oil was added, and 40 amplification cycles (1 minute at 94°C, 1 minute 30 seconds at 55°C, and 1 minute at 74°C each) were performed. The amplification products were analyzed by 2% agarose gel electrophoresis and visualized with UV after ethidium bromide staining. Methods for both amplification rounds of the nested PCR were the same as for the single-round PCR. To measure HEV concentrations in the pooled fecal pig farm samples, end-point dilution PCR was performed with the US-2 sample as a reference.

### Southern Blot Hybridization

RT-PCR was followed by Southern blot hybridizations. An HEV-specific probe was developed based on the consensus sequence of RT-PCR products of the human and swine HEV control samples. The probe sequence was 5'gagaatgcdcagcaggayaaggg3'. For Southern blotting, the RT-PCR products in the agarose gel were denatured by incubating in 0.5 M NaOH for 30 minutes and transferred to a positively charged nylon membrane (Boehringer, Almere, Netherlands) by vacuum blotting (Millipore, Etten-Leur, Netherlands).

Hybridization of HEV RT-PCR products was performed as described for *Norwalk-like virus* by Vinjé et al. (14). Briefly, the nylon membranes were prehybridized for 30 minutes at 42°C in 20 mL 2x SSPE (300 mM NaCl, 20 mM $NaH_2PO_4H_2O$, 2 mM EDTA, pH 7.4) with 0.1% sodium dodecyl sulfate (SDS). The membranes were left for 45 minutes at 42°C to allow hybridization, after addition of 40 pmol of each of the 5'-biotinylated probes (14). The membranes were washed three times for 10 minutes at 42°C with 2x SSPE and 0.1% SDS. Then the membranes were incubated

Table 1. Oligonucleotide primers employed for RT-PCR amplification

| Primer | Sense | Sequence (5' to 3') | Position in genome | Reference |
|---|---|---|---|---|
| ORF1-s1 | Sense | Ctggcatyactactgcyattgagc | 56-79 | 13 |
| ORF1-a1 | Antisense | Ccatcrarrcagtaagtgcggtc | 451-473 | 13 |
| ORF1-s2 | Sense | Ctgccytkgcgaatgctgtgg | 104-124 | 12 |
| ORF1-a2 | Antisense | Ggcagwrtaccarcgctgaacatc | 367-389 | 12 |
| ORF2-s1 | Sense | Gacagaattratttcgtcggctgg | 6298-6321 | 13 |
| ORF2-a1 | Antisense | Cttgttcrtgytggttrtcataatc | 6470-6494 | 13 |
| 3156-EF | Sense | Aaytatgcmcagtaccgggttg | 5687-5708 | 7 |
| 3157-ER | Antisense | Cccttatcctgctgagcattctc | 6395-6417 | 7 |
| 3158-EF | Sense | Gtyatgytyygcatacatggct | 5972-5993 | 7 |
| 3159-IRS | Antisense | Agccgacgaaatyaattctgtc | 6298-6319 | 7 |

[a]Nucleotide positions are numbered according to the Burmese strain (10).

with 1:4,000 diluted streptavidin-peroxidase conjugate (Boehringer, Almere, the Netherlands) for 45 minutes at 42°C in 10 mL of 2x SSPE and 0.5% SDS. After washing three times (10 minutes each) with decreasing concentrations of SDS (0.5%, 0.1%, and 0%) in 2x SSPE, the membranes were incubated for 2 minutes with the enhanced chemoluminescence (ECL) detection reagents (Amersham Life Science, s'Hertogenbosch, the Netherlands), and then were exposed to an ECL hyperfilm (Amersham Life Science) for 30 minutes and overnight to visualize the bound probe.

### Cloning, Sequence Comparison, and Phylogenetic Analysis

HEV RT-PCR products of expected sizes from the pig farm samples were excised from a 2% agarose gel, purified with a Qiaquick gel extraction kit (Qiagen, Hilden, Germany), and cloned into pGEM T-Easy Vector System II (Promega, Madison, WI). After transformation, five positive colonies of each ligation were selected. The pGEM T-Easy Vector was checked for correct insertion size by direct PCR amplification with M13 forward and M13 reverse primers. Correct PCR products were purified with PCR purification kit (Qiagen) and sequenced with the BigDye Terminator Cycle Sequencing Ready Reaction Kit (Perkin Elmer, Applied Biosystems, Foster City, CA) by the use of PCR primers. Nucleotide sequences were edited by using Seq Ed (V1.03, Applied Biosystems) and aligned by Bionumerics (V2.0 Applied Maths, Kortrijk, Belgium). Distance calculations were done by the Jukes and Cantor correction for evolutionary rate (15). The confidence values of the internal nodes were calculated by performing 100 bootstrap analyses. Evolutionary trees for nucleotide sequences were drawn by the Jukes and Cantor method, with HEV strain Burma (GenBank accession number M73218) bp 125-366 and bp 5,994-6,297 as reference.

### Electron Microscopy

Electron microscopy procedures were performed as recommended by Flewett (16) and Doane and Anderson (17). Briefly, a 10% fecal suspension in phosphate-buffered saline was clarified by centrifugation for 30 minutes at 3,000 x *g* at 4°C. The supernatant fluid was collected and centrifuged for 1 hour at 90,000 x *g* at 4°C. The pellet was resuspended in 1 drop of distilled water, and the grids were negatively stained with 2% K-phosphotungstic acid (pH 7.0). Grids were investigated for the presence of viruses with an electron microscope, model Philips 400T (Philips, Eindhoven, the Netherlands) at 80 kV. Identification of virus particles was based on morphologic criteria, i.e., size and characteristic surface morphology (18). The diameters of the virus particles were measured directly on the negatives, instead of on the prints. A 10x measuring magnifier with metric scale was used for particle measurements. Magnification calibration was performed each year with a crossed-line grating replica. All fecal swine farm samples (n = 115) were screened by electron microscopy for viruses.

### Results

### RNA Detection and Virus Detection

In 20 of the pooled samples from the swine farms, HEV was detected by single-round RT-PCR with the primer pair ORF2-s1/ORF2-a1. None of the nine individual samples from pigs with diarrhea contained HEV RNA by RT-PCR. Southern blot hybridization with a probe designed for both human and swine HEV strains confirmed all RT-PCR-positive reactions and identified 5 more positive pooled samples, for a total of 25 (22%) of 115 HEV-positive farm samples.

Nested RT-PCR of ORF1 and ORF2 fragments, with different primer sets, was performed for sequencing. Nested RT-PCR of the 25 HEV-positive samples with the primer sets targeting ORF1 resulted in PCR products of specific size in 18 samples. Nested RT-PCR with the primer sets targeting ORF2 resulted in PCR products of specific size in 17 samples. Five of these samples were positive in the first amplification round. In one sample, our single-round screening RT-PCR was negative, but the nested RT-PCR was positive.

PCR titers (endpoint dilution PCR) of the pooled fecal pig farm samples were between 10 E.4 and 10 E.2. In comparison, the US-2 isolate had an infectivity titer of approximately 10 E.6 and reached 10 E.2 positive dilutions by PCR (Figure 1).

Electron microscopy analysis of the 25 RT-PCR positive samples revealed particles with HEV-like morphologic features in only one pig farm sample. The diameter of these particles was 31.5 nm.

### Cloning, Sequence Comparison, and Phylogenetic Analysis

For 14 HEV isolates, nucleotide sequences of both ORF1 and ORF2 PCR products were obtained. Cloned sequences from the same sample showed little or no diversity in ORF1 as well as ORF2 fragments. Only cloned sequences were used in the phylogenetic analyses. The sequences reported in this paper have been deposited in GenBank (accession numbers AF336290-336299 and AF335998-336014). Comparison of the nucleotide sequences showed percent nucleic acid identities of 82.0% to 95.5% in the 242-bp fragment of ORF1 and 79.5% to 92.7% in the 304-bp fragment of ORF2 among swine HEV isolates from the Netherlands. The comparative analysis of sequences of the capsid encoding region ORF2 from GenBank indicated that parts of the Dutch swine sequences (NLSW22 and NLSW122) were closely related (90.0% to 90.9%) to the U.S. human and swine strains, and

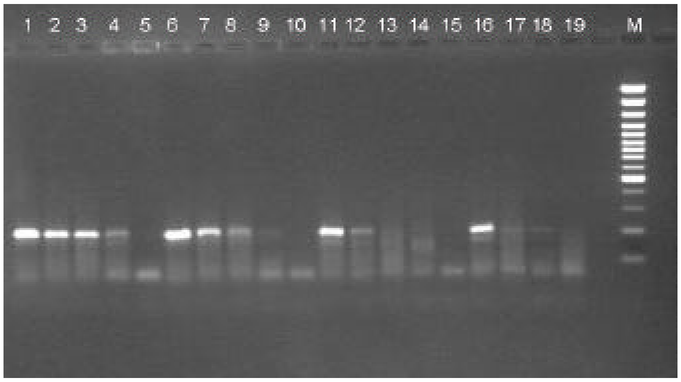

Figure 1. Endpoint dilution polymerase chain reaction with the US2 human *Hepatitis E virus* (HEV) sample as reference (infectivity titer approximately 10 E.6). M: molecular mass marker; lanes 1-4 NLSW50 $10^{-1}$,$10^{-2}$,$10^{-3}$ ;lanes 6-9: NLSW15 $10^{-1}$,$10^{-2}$,$10^{-3}$; lanes 11-14: NLSW20 $10^{-1}$,$10^{-2}$,$10^{-3}$; lanes 16-19 US2 $10^{-1}$,$10^{-2}$,$10^{-3}$. Lanes 5,10 and 15 negative control water samples.

others (NLSW50) were closely related (91.8% to 93.1%) to human and swine strains from Spain (Table 2). Comparison with other isolates from endemic areas showed a nucleotide identity <79.8% in both fragments. Many of these changes did not result in differences at the amino acid level.

By phylogenetic analysis, the swine HEV sequences of ORF1 and ORF2 formed at least two separate clusters. Seven of 14 Dutch isolates were closely related to the U.S. human and swine isolates. The other seven Dutch isolates were closely related to European HEV isolates from humans and swine (Figures 2 and 3).

### Discussion

To determine whether HEV is prevalent in swine in the Netherlands, we used RT-PCR methods, with primers located in the HEV ORFs 1 and 2. Cloned sequences of the PCR product showed little or no diversity, suggesting that only one or a few HEV strains circulated in a pig farm. Despite the fact that PCR titers revealed reasonable quantities of virus (Figure 1), particles with HEV-like morphologic features could be detected by electron microscopy in only one of the 25 RT-PCR positive samples. This relatively low number of positives by electron microscopy can be explained by the greater sensitivity of RT-PCR and may also have resulted from freeze-thawing the samples. HEV-like caliciviridae have been described as sensitive to freeze-thawing (27).

This is the first report with direct evidence of HEV in swine in Europe. Pina et al. (21) reported detection of HEV sequences in sewage from a swine slaughterhouse, suggesting that HEV might be present in swine. The rather high prevalence of HEV in commercial swine farms suggests that it is widespread in the general swine population. In this study, clinical symptoms in swine were not recorded, and no overt clinical symptoms were observed. Therefore, a clinical association with HEV infection could not be demonstrated.

Table 2. Nucleotide identity (%) between NLSW *Hepatitis E virus* (HEV) isolates, with respect to other HEV strains, in sections of 242 bases of ORF1 and 304 bases of ORF2[a]

| | ORF1 fragment | | | | ORF2 fragment | | | |
|---|---|---|---|---|---|---|---|---|
| HEV strain | NLSW15 | NLSW22 | NLSW50 | NLSW105 | NLSW15 | NLSW22 | NLSW50 | NLSW105 |
| NZ1 | 82.0 | 85.7 | 82.3 | 84.2 | Na[b] | Na | Na | na |
| Italy | 80.8 | 82.3 | 80.8 | 83.8 | Na | Na | Na | na |
| Arg1 | 82.7 | 85.7 | 83.5 | 88.4 | Na | Na | Na | na |
| Arg2 | 79.5 | 83.3 | 81.0 | 84.8 | Na | Na | Na | na |
| US1 | 80.5 | 92.9 | 82.7 | 87.2 | 86.7 | 90.9 | 83.9 | 86.4 |
| US2 | 80.8 | 91.4 | 83.1 | 85.7 | 85.7 | 90.9 | 83.6 | 86.7 |
| USswine | 81.2 | 87.6 | 83.5 | 84.2 | 86.1 | 90.0 | 83.0 | 85.7 |
| NLSW15 | 100.0 | 82.0 | 94.7 | 83.8 | 100.0 | 83.8 | 90.9 | 82.7 |
| NLSW22 | 82.0 | 100.0 | 84.2 | 87.6 | 83.8 | 100.0 | 83.3 | 85.7 |
| NLSW50 | 94.7 | 84.2 | 100.0 | 85.7 | 90.9 | 83.3 | 100.0 | 84.4 |
| NLSW105 | 83.8 | 87.6 | 85.7 | 100.0 | 82.7 | 85.7 | 84.4 | 100.0 |
| NLSW36 | 84.2 | 86.1 | 85.3 | 91.0 | 83.0 | 84.7 | 83.2 | 89.4 |
| NLSW82 | 93.2 | 85.3 | 93.2 | 83.8 | 91.0 | 79.5 | 91.2 | 83.3 |
| NLSW85 | 91.7 | 85.3 | 93.6 | 86.1 | 91.3 | 92.5 | 92.7 | 84.2 |
| NLSW122 | 82.7 | 95.5 | 83.5 | 88.4 | 83.2 | 82.6 | 83.8 | 86.0 |
| Greece1 | 92.1 | 82.3 | 92.9 | 83.8 | Na | Na | Na | Na |
| Greece2 | 85.7 | 80.8 | 83.1 | 79.3 | Na | Na | Na | Na |
| Egypt | Na | Na | Na | Na | 75.6 | 76.5 | 76.5 | 76.5 |
| Morocco | Na | Na | Na | Na | 77.5 | 77.2 | 78.1 | 76.5 |
| VH1 | 92.1 | 83.5 | 92.1 | 83.8 | 91.1 | 80.9 | 93.1 | 82.9 |
| VH2 | 91.0 | 83.1 | 91.7 | 83.5 | 92.4 | 80.3 | 92.4 | 81.6 |
| E11 | Na | Na | Na | Na | 92.8 | 80.9 | 91.8 | 82.6 |
| Barcelona | 79.3 | 73.7 | 78.2 | 75.9 | 76.9 | 76.2 | 77.8 | 79.0 |
| Nepal | 79.3 | 73.7 | 78.2 | 75.9 | 78.1 | 78.1 | 79.6 | 79.3 |
| Burma1 | 79.7 | 72.9 | 78.6 | 76.3 | 76.9 | 76.8 | 78.4 | 78.1 |
| India1 | 79.0 | 73.7 | 78.6 | 75.6 | 78.7 | 76.5 | 79.0 | 78.7 |
| China1 | 79.0 | 72.9 | 78.6 | 75.6 | 77.5 | 77.8 | 78.4 | 78.4 |
| Pakistan | 79.3 | 73.3 | 78.2 | 75.9 | 77.5 | 77.2 | 78.4 | 77.5 |
| New China(T1) | 80.5 | 77.8 | 79.7 | 78.6 | 79.0 | 79.6 | 78.7 | 77.2 |
| Mexico | 78.2 | 77.8 | 77.5 | 76.3 | 76.0 | 75.4 | 76.0 | 75.1 |

[a]For explanation of isolates' acronyms, see Figure 2 legends.
[b]na= not available

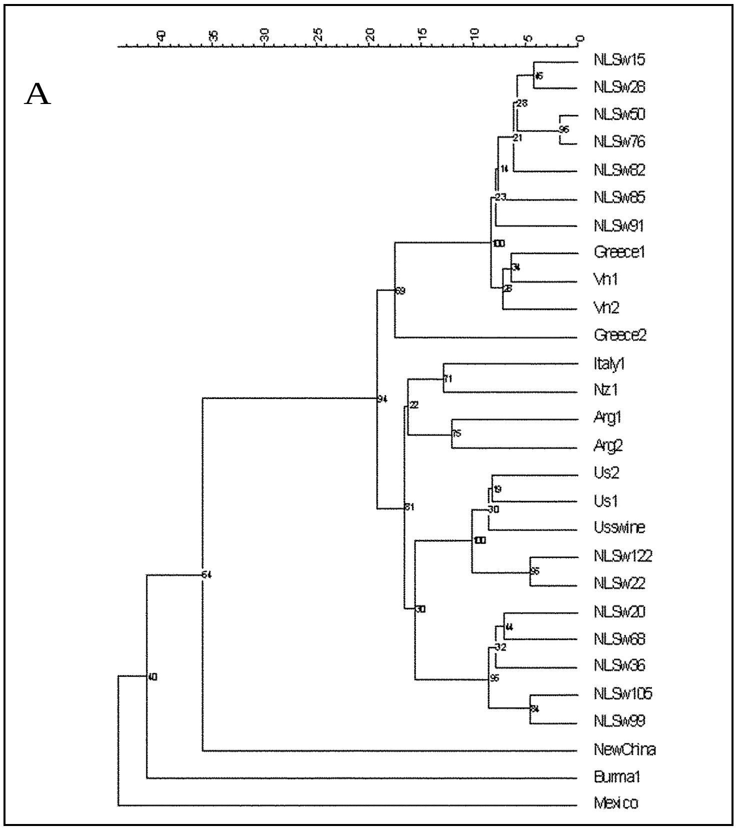

Other studies with different designs will be needed to find out whether HEV can cause clinical disease in swine. HEV may run a subclinical course in swine (7), a situation resembling the mostly asymptomatic hepatitis A and E infections in children (4). The outcome of natural HEV infection in adult and pregnant pigs is unknown and needs to be evaluated. In addition, it is unknown whether subclinical HEV infection may have adverse effects on growth rates in juvenile pigs.

On the basis of sequence comparisons, genetic distances, and phylogenetic analyses of the 242 bases of ORF1 and the 304 bases of ORF2, all swine HEVs in the Netherlands clustered with previously described European or American human or swine HEV isolates. There appears to be geographic clustering of swine and human sequences in Europe, America, and Asia. Only one Asian isolate (from New Zealand) clustered with a European isolate. The observation that American, European, and Asian human and swine isolates group together suggests relatively recent interspecies transmission in different parts of the world. To determine whether human HEV evolved from swine HEV or vice versa, the retrospective studies of archived fecal or serum samples from humans and swine may provide information about the evolutionary relationship between swine and human HEV.

An important issue raised from the discovery of swine HEV strains similar to human strains is the possibility of actual zoonotic transmission from swine to

Figure 2. Phylogenetic relationships among human and pig strains of *Hepatitis E virus* (HEV), based on a 242-bp sequence of ORF1 (nucleotides 125-366). Rooted tree (A) and unrooted tree (B). In the rooted tree, all the Dutch swine sequences are depicted with the foreign isolates that cluster with those sequences, as well as prototype isolates from different clusters. The distances can be estimated by using the scale, and the numbers are confidentiality rates. In the unrooted tree, eight Dutch swine sequences selected on the basis of diversity are depicted with isolates from different geographic origins. The numbers correspond with distances. NLSw15, 20, 22, 28, 36, 50, 68, 76, 82, 85, 91, 99, 105, and 122: 14 Dutch pig HEV. US1, US2 and USswine: United States human and pig HEV strains, respectively (19). Greece 1 and Greece 2: Greek human HEV strains (13). Arg1 and Arg 2: Argentinian human isolates (20). VH1 and VH2: Spanish human isolates; and E11: Spanish slaughterhouse sewage isolate (21). Burma1: Burmese human isolate (22). Italy1: human isolate (13). Barcelona: sewage isolate (23). Pakistan: human isolate (24). China1: human isolate (25). India1: human isolate (GenBank acc. no. X99441). NZ1: New Zealand human isolate (GenBank acc. no AF215661). Nepal: human isolate (26). Egypt: human isolate (22). Morocco: human isolate (7). NewChina (T1):GenBank acc. No AJ272108).

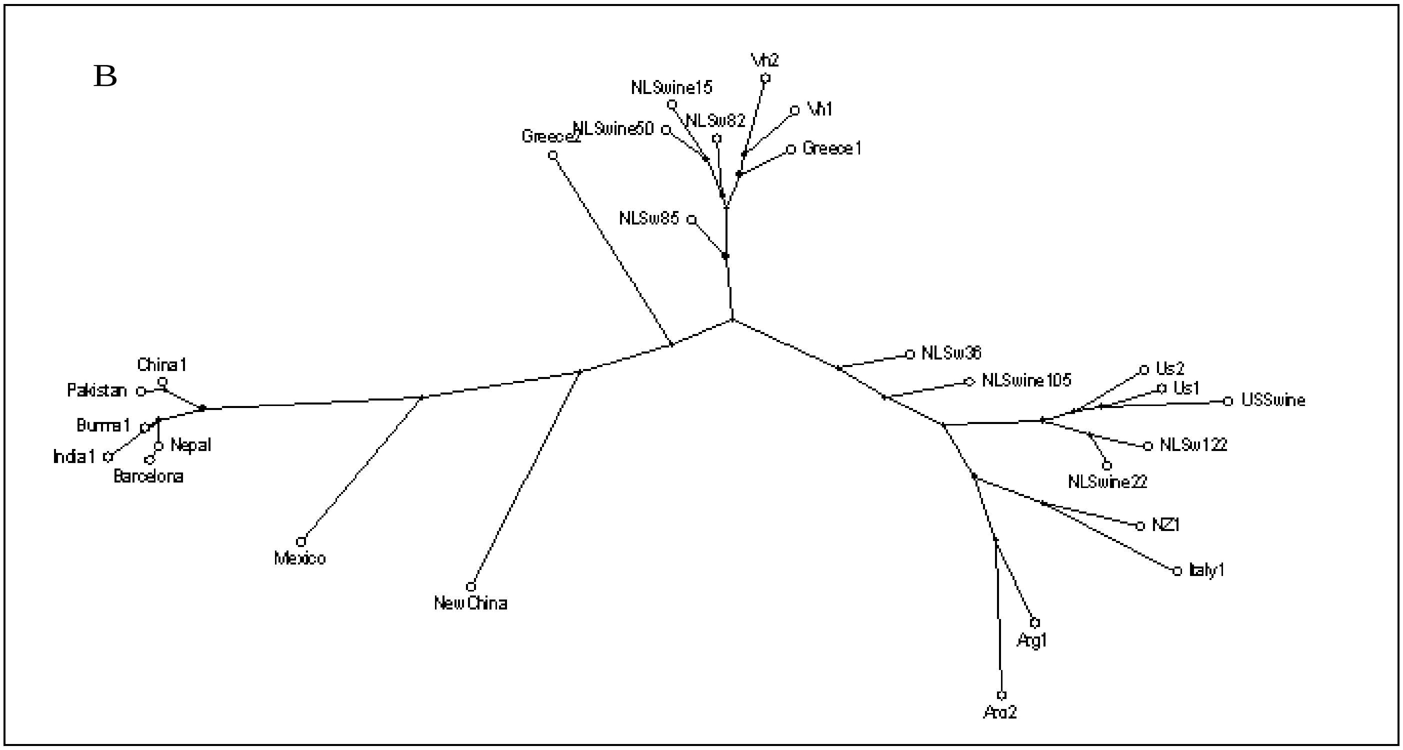

humans. Based on the sequence similarities observed among the Dutch swine HEV strains and the European and North American human strains, one cannot yet determine whether these swine strains are species-specific or circulating in the human population as well. Swine may be a reservoir for human infection. A reported higher anti-HEV seroprevalence among pig farmers working in close contact with pigs versus persons whose work does not involve contact with livestock (28) suggests that swine HEV may infect humans. If zoonotic HEV infections occur, whether HEV from swine can cause clinical disease in humans warrants study. Clinical HEV infection in the Netherlands in persons without a history of travel has not yet been observed; however, nontravelers with hepatitis are seldom tested for HEV.

HEV has been detected in sewage in Spain (23). The discovery of HEV in swine in the Netherlands suggests that humans may become infected by contact with sewage of animal origin or even through contact with surface waters.

In addition to the public health concern about zoonosis, there is also the concern for xenozoonosis, the inadvertent transmission of pathogens from animal organs to human recipients. Nonpathogenic pig HEV strains may become pathogenic for humans after xenotransplantation, as a result of species jumping, recombination, or adaptation in immunocompromised xenotransplantation recipients (29).

In conclusion, the discovery of swine HEV strains in

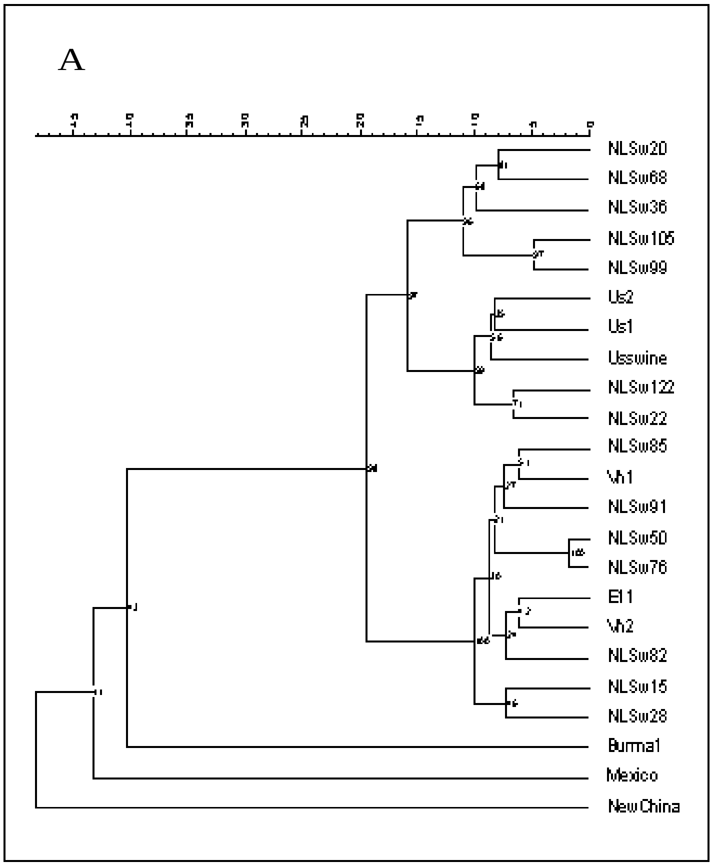

Figure 3. Phylogenetic relationships among human and pig *Hepatitis E virus* strains, based on 304 nucleotide sequences of HEV ORF2 (nucleotides 5994-6297). Rooted tree (A) and unrooted tree (B). For further explanation of the figure and definition of isolate acronyms, see Figure 2 legend.

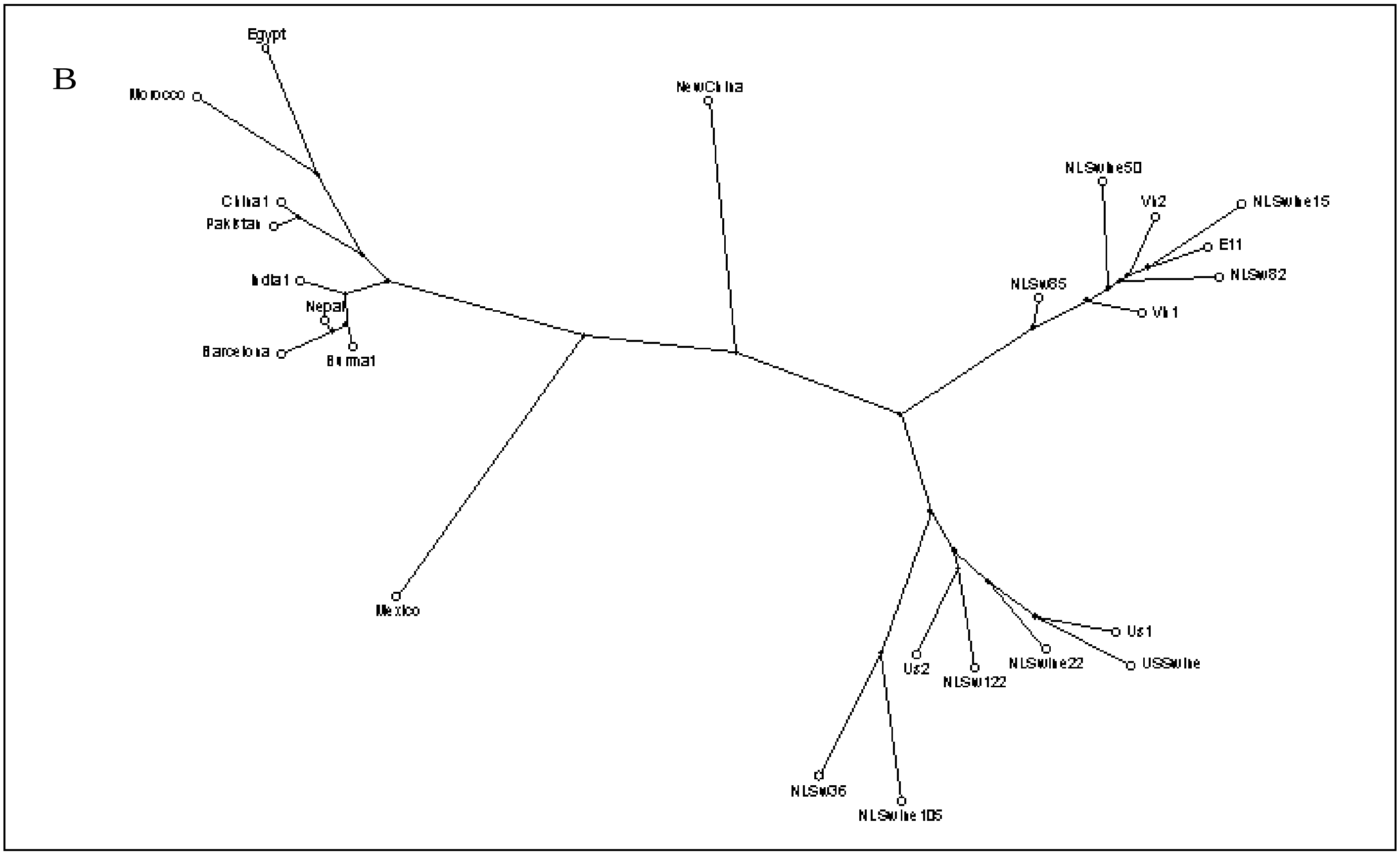

the Netherlands related to human HEV isolates from Europe and America indicates an important new direction for HEV research. From the public health point of view, methods should be developed to detect interspecies transmission at an early stage. Swine HEV infection may provide an animal model for HEV studies, and swine HEV might also prove useful for development of a vaccine against human hepatitis E.

### Acknowledgments

We thank M. Favorov and R. Purcell for providing the HEV-positive control samples, K. Peperkamp for providing the samples from pigs with diarrhea, and A.W. van de Giessen and W.D.C. Deisz for making fecal specimens available from the monitoring study of zoonotic enteric pathogens.

This research was financially supported and approved by the Dutch Ministry of Public Health, Welfare and Sports.

Dr. van der Poel is a veterinary virologist in the Microbiological Laboratory for Health Protection, National Institute of Public Health and the Environment the Netherlands. His research involves viral zoonoses and foodborne virus infections.

### References

1. Berke T, Matson DO. Reclassification of the Caliciviridae into distinct genera and exclusion of hepatitis E virus from the family on the basis of comparative phylogenetic analysis. Arch Virol 2000:145:1421-36.
2. Zaaijer HL, Kok M, Lelie PN, Timmerman RJ, Chau K, van der Pal HJ. Hepatitis E in The Netherlands: imported and endemic. Lancet 1993;341:826.
3. Aggarwal R, Krawczynski K. Hepatitis E: an overview and recent advances in clinical and laboratory research. J Gastroenterol Hepatol 2000;15:9-20.
4. Aggarwal R, Kini D, Sofat S, Naik SR, Krawczynski K. Duration and faecal viral excretion in acute hepatitis E. Lancet 2000;356:1081-2.
5. Balayan MS, Usmanov RK, Zamyatina NA, Djumalieva DI, Karas FR. Experimental hepatitis E infection in domestic pigs. J Med Virol 1990;32:58-9.
6. Clayson ET, Innis BL, Myint KS, Narupiti S, Vaughn DW, Giri S, et al. Detection of hepatitis E virus infections among domestic swine in the Kathmandu Valley of Nepal. Am J Trop Med Hyg 1995;53:228-12.
7. Meng XJ, Purcell RH, Halbur PG, Lehman JR, Webb DM, Tsareva TS, et al. A novel virus in swine is closely related to human hepatitis E virus. Proc Natl Acad Sci U S A 1997;94:9860-5.
8. Noordhuizen JP, Frankena K. *Salmonella* enteritidis: clinical epidemiological approaches for prevention and control of *S.* enteritidis in poultry flocks: a basic approach. Int J Food Microbiol 1994;21:131-43.
9. Boom R, Sol CJA, Salimans MMM, Jansen CL, Wertheim-van Dillen PM, van der Noordaa J. Rapid and simple method for purification of nucleic acids. J Clin Microbiol 1990;28:495-503.
10. Huang RT, Li DR, Wei J, Huang XR, Yuan XT, Tian X. Isolation and identification of hepatitis E virus in Xinjang, China. J Gen Virol 1992;73:1143-8.
11. Kwok S, Higuchi R. Avoiding false positives with PCR. Nature 1989;339:237-8.
12. Wang Y, Ling R, Erker JC, Zhang H, Li H, Desai S, et al. A divergent genotype of hepatitis E virus in Chinese patients with acute hepatitis. J Gen Virol 1999;80:169-77.
13. Schlauder GG, Desai SM, Zanetti AR, Tassopoulos NC, Mushahwar IK. Novel hepatitis virus (HEV) isolates from Europe: evidence for additional genotypes of HEV. J Med Virol 1999;57:243-51.
14. Vinjé J, Altena SA, Koopmans MPG. The incidence and genetic variability of small round-structured viruses in outbreaks of gastroenteritis in The Netherlands. J Infect Dis 1997;176:1374-8.
15. Van der Peer Y, De Wachter R. TREECON for windows: a software package for the construction and drawing of evolutionary trees for the Microsoft Windows environment. Computer Applications in Bioscience 1994;10:569-70.
16. Flewett TH. Electron microscopy in the diagnosis of infectious diarrhea. J Am Vet Med Assoc 1978;173:538-43.
17. Doane FW, Anderson N. Pretreatment of clinical specimens and viral isolates. In: Electron microscopy in diagnostic virology. Cambridge: Cambridge University Press; 1987. p. 4-10.
18. Caul EO, Appleton H. The electron microscopical and physical characteristics of small round human fecal viruses: an interim scheme for classification. J Med Virol 1982;9:257-65.
19. Erker JC, Desai SM, Schlauder GG, Dawson GJ, Mushawar IK. A hepatitis E virus variant from the United States: molecular characterization and transmission in cynomolgus macaques. J Gen Virol 1999;80:681-90.
20. Schlauder GC, Frider B, Sookoian S, Castario GC, Mushawar IK. Identification of 2 novel isolates of hepatitis E virus in Argentina. J Infect Dis 2000;182:294-7.
21. Pina S, Buti M, Cotrina M, Piella J, Girones R. HEV identified in serum from humans with acute hepatitis and in sewage of animal origin in Spain. J Hepatol 2000;33:826-33.
22. Tam AW, Smith MM, Guerra ME, Huang CC, Bradley DW, Fry KE, et al. Hepatitis E virus (HEV) molecular cloning and sequencing of the full length viral genome. Virology 1991;185:120-31.
23. Pina S, Jofre J, Emerson SU, Purcell RH, Girones R. Characterization of a strain of infectious hepatitis E virus isolated from sewage in an area where hepatitis E is not endemic. Appl Environ Microbiol 1998;64:4485-90.
24. Tsarev SA, Emerson SU, Tsareva TS, Yarbough PO, Lewis M, Govindarajan S, et al. Phylogenetic analysis of hepatitis E virus isolates from Egypt. J Med Virol 1999;57:68-74.
25. Yin S, Tsarev SA, Purcell RH, Emerson SU. Partial sequence comparison of eight new Chinese strains of hepatitis E virus suggests that the genome sequence is relatively stable. J Med Virol 1993;41:230-41.
26. Gouvea V, Snellings N, Popek MJ, Longer CF, Innis BL. Hepatitis E virus: complete genome sequence and phylogenetic analysis of a Nepali isolate. Virus Res 1998;57:21-6.
27. Bradley DW. Hepatitis E: epidemiology, aetiology and molecular biology. Med Virol 1992;2:19-28.
28. Drobeniuc J, Favorov MO, Shapiro CN, Bell BP, Mast EE, Dadu A, et al. Hepatitis E virus antibody prevalence among persons who work in close contact with pigs. J Infect Dis 2001. In press.
29. Murphy FA. The public health risk of animal organ and tissue transplantation into humans. Science 1996;273:746-7.
